# Recall of physical activity advice was associated with higher levels of physical activity in colorectal cancer patients

**DOI:** 10.1136/bmjopen-2014-006853

**Published:** 2015-04-27

**Authors:** A Fisher, K Williams, R Beeken, J Wardle

**Affiliations:** Department of Epidemiology and Public Health, University College London, London, UK

**Keywords:** Physical activity, Oncology, Lifestyle, PROMS

## Abstract

**Objectives:**

The present study tested the hypothesis that recall of receiving physical activity (PA) advice would be associated with higher levels of PA in patients with a diagnosis of colorectal cancer (CRC).

**Setting:**

Colorectal cancer patients who were diagnosed in 2010 or 2011, and had been treated in the English National Health Service (NHS).

**Participants:**

17 753 respondents completed at least one section of the survey relevant to the current study and after exclusion of 171 with dementia (since results relied on recall), 15 254 had complete data for the current study. 60% were male, 67% were >65 years and 96% were from a white ethnic group.

**Primary and secondary outcome measures:**

Patients completed the ‘Living with and Beyond Colorectal Cancer’ Patient-Reported Outcome Measures (PROMS) survey in 2013. The survey included questions on receiving exercise advice/information (‘PA advice’), and the frequency of currently doing at least 30 min of brisk PA per day (‘PA level’: 0, 1–4 or 5–7 days, within the past week; with the top category meeting UK guidelines).

**Results:**

A third of respondents (31%) recalled receiving PA advice. Independent of demographics and treatment, patients who recalled having PA advice were more likely to be currently doing some brisk PA (51% in the advice group vs 42% in the no advice group; OR 1.74, 95% CI 1.60 to 1.90; p<0.001), and more likely to be meeting PA guidelines (25% vs 20%; OR 1.70, CI 1.54 to 1.88; p<0.001).

**Conclusions:**

Recalling being given PA advice after a diagnosis of CRC was associated with higher levels of PA. However, less than a third of patients recalled receiving advice. Future research should examine the context in which advice is given and randomised trials are required. However, encouraging clinicians working with patients with CRC to give brief PA advice is warranted and may help improve outcomes for CRC survivors.

**Strengths and limitations of this study**

- This is the first very large population-based study to explore whether physical activity advice is given to colorectal cancer patients; proportions who recalled receiving advice were low.
- Physical activity advice during the cancer care pathway was associated with higher levels of physical activity.
- The findings of this study provide strong impetus to clinicians working with colorectal cancer to recommend physical activity, a practice which does not yet appear to be routine in the UK.
- This study is limited in relying on patient report of physical activity levels and recall of whether they were given advice. However, the Patient-Reported Outcome Measures data are designed to collect data on patient experience on a large scale.
- Data on the context of the advice were not available and this should be a focus of future research.

## Introduction

There is now a solid body of evidence that a physically active lifestyle is associated with better long-term outcomes following a diagnosis of colorectal cancer (CRC).^1–3^ A meta-analysis of 7 prospective cohort studies demonstrated that higher postdiagnosis physical activity (PA) was associated with lower CRC-specific and all-cause mortality.^1^ A report from the Cancer Prevention Study-II (CPS-II) Nutrition Cohort showed associations with all-cause mortality for both postdiagnosis PA and sedentary time.^2^ PA has also been associated with better quality of life and lower levels of cancer-related fatigue.^3^

CRC survivors typically have lower levels of PA than the general population^4^ and data from the English Longitudinal Study of Ageing showed a faster decline in activity over time in participants who received a cancer diagnosis than the rest of the cohort.^5^ Intervention within the clinical care pathway to promote PA could help CRC survivors achieve the health gains associated with an active lifestyle.

Clinicians dealing with patients with cancer are in a good position to offer activity advice, and they are the patients’ preferred source of information.^6^ Patients also specifically express a wish for those involved in their care to initiate discussion about PA during consultation.^7^ However, evidence to date indicates that most patients with cancer are not receiving PA advice. In a US survey, conducted in 2008, 38% of oncologists and surgeons said they did not enquire about patients’ activity levels.^8^ A similar study in the UK found that 56% of healthcare professionals did not discuss PA with their patients with breast cancer^9^ and a more recent survey suggested little improvement in these figures.^10^ These findings are broadly consistent with reports from cancer survivors themselves, with only a third of patients with breast or prostate cancer reporting being given any PA advice.^11^ Primary care physicians are another source of lifestyle advice, but although US data indicate some increase in provision of PA advice for patients with cancer in primary care (25% in 2000, 36% in 2010), the rates are still too low.^12^

Brief PA advice in primary care has been shown to increase PA in sedentary adults^13^ and a review concluded that it was an effective intervention, at least in the short-term.^14^ Studies in cancer survivors also indicate that clinicians discussing PA with their patients might have a positive effect on activity levels. A survey of 311 cancer survivors (38 with CRC), found that oncologist-initiated discussion of PA was associated with higher activity levels during treatment^7^ and an exploratory study in 24 breast cancer survivors suggested that a clinician recommendation was an important factor in exercise adherence.^15^ In one of the few trials of brief advice in cancer survivors, a simple PA recommendation doubled the percentage of breast cancer survivors meeting national exercise guidelines, with stronger effects among those who correctly recalled the advice, although the follow-up interval in this study was only 5 weeks.^16^

The current study used data collected from a very large sample of patients with CRC to test the hypothesis that recalling being given PA advice would be related to higher levels of physical activity.

## Materials and methods

### Participants and measures

Data were from the *Living with and Beyond Colorectal Cancer* survey commissioned by the UK Department of Health in 2013, as part of a programme of work designed to ensure that the needs of patients with colorectal cancer in the UK are met across a range of health, psychosocial and lifestyle domains through the recording of patient-reported outcomes (PROMS).^17^ The questionnaire was mailed by the National Cancer Registration Service (NCRS), in 2013, to a sample of 34 467 adult patients with a recorded diagnosis of CRC in 2010 or 2011, who were treated in the English National Health Service, and who were >16 years and were 12–36 months postdiagnosis. Content and face validity for the PROMS survey were identified through expert reviews and consultations with patients, experts and charity advisory groups.^18^ For the current study, a formal request was made by the study authors to the NCRS for health and lifestyle data for the secondary analyses.

The questionnaire section ‘Overall Support and Care’ included the question ‘*Did you receive any advice or information on physical activity and exercise* (‘physical activity advice’)?’ This was within a longer list of cancer-relevant domains of information, including physical and psychological aspects of living with cancer, finance, employment, family and support services.

Current PA was assessed by asking: ‘In the past week how many days have you done 30 min or more of brisk physical activity (This may include sport, exercise or brisk walking or cycling for recreation or to get to and from places, but should not include housework that or physical activity that is part of your job)?’ Responses were categorised as ‘none’ (0 days), ‘some’ (1–4 days) or ‘meeting guidelines’ (5–7 days), broadly in line with the UK Government recommendations for the general population^19^ and the American College of Sports Medicine recommendations that cancer survivors should participate in at least 150 min per week of at least moderate activity.^20^ There were also data on sex and age at diagnosis (obtained from registry data), and ethnicity (reported by patients). Index of Multiple Deprivation (IMD) scores were calculated for the home postcode (1–5 from least to most deprived).^21^ IMD is an area level measure derived from a composite of 38 indicators across seven domains of deprivation, including income, employment, health, education, housing and services, living environment and crime.^21^

There were a variety of treatment questions, but for these analyses we included: ‘*How has your CRC responded to treatment*?’ (‘fully responded I am in remission’, ‘has been treated but is still present’, ‘has not been treated’, ‘has come back after initial treatment’, ‘not certain what is happening’). Length of time since treatment was also reported. Long-term health conditions (LTCs) were assessed with ‘*Do you have a long-standing health condition other than cancer*?’ *(*‘yes’, ‘no’, ‘can't say’). As the focus of this analysis was on *recall* of receiving PA advice, patients reporting a diagnosis of dementia were excluded. The full survey is available at cancerproms.ncr.nhs.uk. In accordance with UCL Ethics Committee guidance, additional ethical approval was not required for secondary analyses of anonymous health surveillance survey data.

## Analyses

Where >75% of respondents fell into a single response category, predictor variables were dichotomised for analyses. However, full descriptive data, including the proportion of missing values, are presented in the results. Ethnic groups were categorised as ‘white’ versus ‘all other’. Since health and treatment variables were secondary to the main research question, missing data were recoded as ‘unknown’ for analyses to include as many respondents as possible. Response to treatment was categorised as ‘in remission’ versus ‘all other’. Time since treatment was coded as ‘Still having treatment’, ‘<1 year’ post-treatment, ‘>1 year’, or ‘unknown’. Presence of an LTC was recorded as ‘yes’, ‘no’, or ‘unknown’.

Descriptive statistics and percentages in each category were calculated. Two types of logistic regression were carried out. First, to assess factors associated with whether PA advice was given, a binary logistic regression with ‘advice’ or ‘no advice’ as the outcome was carried out. Second, to assess whether advice related to activity levels, a multinomial logistic regression model with PA level (none/some/meeting activity guidelines) as the outcome was carried out. In each case, simple associations and then models adjusted for potential confounders were presented. Analyses were carried out in SPSS V.18. Given the large sample size, significance was set at p<0.01.

## Results

Of the 34 467 questionnaires mailed out by NCRS, 21 802 (63.3%) were returned at least partially completed and further information on this sample are provided in a recent paper by Downing *et al*.^22^ Regarding the data granted from NCRS for the current study, 17 753 patients had responded to at least 1 question in the relevant parts of the survey. Compared to non-responders, these participants were less likely to be from deprived areas and fewer were from the youngest (<55 years) and oldest (>85 years) age categories (p<0.001). After exclusion of 171 (1.1%) patients with dementia, complete data on the PA questions and demographics were available for 15 254 patients; this constituted the study sample. Characteristics of the 17 753 patients who completed at least 1 question in the relevant parts of the survey and the study sample were very similar, and are shown in table 1.

**Table 1:** Full demographics and treatment variables from responders and study sample

	Responders n=17 753		Study sample n=15 254	
Demographics	n	Per cent	n	Per cent
Sex				
Female	7295	41.1	6091	39.9
Male	10 458	58.9	9163	60.1
Age				
>85	969	5.5	777	5.1
75–84	4508	25.4	3797	24.9
65–74	6195	34.9	5680	37.2
55–64	3712	20.9	3677	24.1
<55	1585	8.9	1323	8.7
Missing	784	4.4	–	–
Ethnicity				
White	17 070	96.2	14 712	96.4
Minority ethnic group	294	1.7	245	1.6
Unknown	389	2.2	297	1.9
IMD category				
5 Most deprived	1947	11.0	1606	10.5
4	2935	16.5	2441	16.0
3	3919	22.1	3335	21.9
2	4419	24.9	3844	25.2
1 Least deprived	4533	25.5	4028	26.4
Physical activity (PA)				
Received PA advice				
Yes	5038	28.4	4734	31
No	11 489	64.7	10 520	69
Missing	1226	6.9	–	–
Activity levels				
None	5803	32.7	5080	33.3
Some	7547	42.5	6877	45.1
Meeting guidelines	3626	20.4	3297	21.6
Missing	777	4.4	–	–
*Treatment and health variables*				
Treatment response				
In remission	13 804	77.8	12 026	78.8
Treated but still present	857	4.8	741	4.9
Has not been treated	159	0.9	123	0.8
Has come back	437	2.5	388	2.5
Not certain	1618	9.1	1341	8.8
Missing	878	4.9	635	4.2
Time since treatment				
Still having	349	2.0	289	1.9
<3 months	173	1.0	139	0.9
3–12 months	2128	12.0	1799	11.8
1–5 years	14 501	81.7	12 598	82.6
>5 years	151	0.9	117	0.8
Do not know	48	0.3	32	0.2
Missing	403	2.3	280	1.8
Long-term condition				
Yes	8225	46.3	7160	46.9
No	6733	37.9	5995	39.3
Do not know	552	3.1	444	2.9
Missing	2243	12.6	1655	10.8

67% were >65 years old, 60% were male, and 96% identified themselves as ‘white’. Most patients reported that their cancer had fully responded to treatment and they were in remission (79%), with smaller numbers reporting that the cancer was still present (5%), had not been treated (1%), had come back after initial treatment (3%) or that they were not certain (9%); however, 4% did not respond to this question. The majority (>80%) of patients were at least 1 year since treatment. Forty-seven per cent reported a long-standing health condition other than cancer, 3% ‘couldn't say’ and 11% did not respond. Forty-five per cent of patients reported doing at least some brisk activity and 22% met the guidelines for PA, but 33% reported doing none.

Overall, 31% of respondents recalled having received any PA advice. The proportion receiving PA advice by demographics, treatment and LTCs is shown in table 2. Men were more likely than women to recall being given advice (35 vs 25%; OR 1.66, 95% CI 1.55 to 1.79; p<0.001). Younger patients were more likely than older patients to recall advice (37% in the <55-year-olds vs 20% in >85-year-olds (OR 2.41, CI 1.95 to 2.90; p<0.001). Patients from higher SES groups were more likely to recall advice than those from lower SES groups (comparing highest to lowest SES, 32% vs 28%; OR 1.25, CI 1.10 to 1.43; p<0.001), but there was no significant association with ethnicity. Patients in remission were more likely to recall being given PA advice (32% vs 27%; OR 1.23, CI 1.12 to 1.30; p<0.001), and in the fully adjusted model, patients with a LTC were statistically more likely to recall advice, but the magnitude of this difference was extremely small (32% vs 31%; p<0.001) and unlikely to be clinically meaningful. Time since treatment was not associated with advice in the adjusted model.

**Table 2:** Associations between being given physical activity (PA) advice, demographic factors, treatment and activity levels 2–3 years later in 15 254 patients with colorectal cancer

	PA advice given n (%)	Model 1 (unadjusted) OR (95% CI)	Model 2 (adjusted) OR (95% CI)
Reference category No PA advice			
Total			
(n=15 254)	4734 (31.0)	–	–
Sex			
Female (n=6091)	1506 (24.7)	1	1
Male (n=9163)	3228 (35.2)	1.66 (1.55 to 1.78)***	1.66 (1.55 to 1.79)***
Age			
>85 (n=777)	156 (20.1)	1	1
75–84 (n=3797)	988 (26.0)	1.40 (1.16 to 1.69)**	1.35 (1.12 to 1.64)***
65–74 (n=5680)	1789 (31.5)	1.83 (1.52 to 2.20)***	1.73 (1.44 to 2.09)***
55–64 (n=3677)	1314 (35.7)	2.21 (1.83 to 2.67)***	2.15 (1.77 to 2.60)***
<55 (n=1323)	487 (36.8)	2.32 (1.88 to 2.86)***	2.41 (1.95 to 2.90)***
IMD			
5 (n=1606)	455 (28.3)	1	1
4 (n=2441)	714 (29.3)	1.05 (0.91 to 1.20)	1.08 (0.94 to 1.24)
3 (n=3335)	1019 (30.6)	1.11 (0.98 to 1.27)	1.16 (1.02 to 1.33)*
2 (n=3844)	1245 (32.4)	1.21 (1.07 to 1.38)**	1.25 (1.10 to 1.43)***
1 (n=4028)	1301 (32.3)	1.21 (1.06 to 1.37)**	1.25 (1.10 to 1.43)***
Ethnicity			
White (n=14 712)	4560 (31.0)	1	1
All other (n=542)	174 (32.1)	1.05 (0.88 to 1.27)	1.03 (0.85 to 1.24)
Time since treatment			
Still having (n=289)	82 (28.4)	1	1
<1 year (n=1938)	597 (30.8)	1.36 (1.03 to 1.79)	1.06 (0.81 to 1.39)
>1 year (n=12 715)	3978 (31.3)	1.39 (1.07 to 1.80)	1.06 (0.80 to 1.41)
Unknown (n=312)	77 (24.7)	1.21 (0.41 to 1.74)	1.04 (0.72 to 1.50)
Treatment response			
All other (n=3228)	868 (26.9)	1	1
In remission (n=12 026)	3866 (32.1)	1.29 (1.18 to 1.41)***	1.23 (1.12 to 1.34)***
Long-term condition			
No (n=5995)	1855 (30.9)	1	1
Unknown (n=2099)	605 (28.8)	0.90 (0.81 to 1.00)	0.98 (0.89 to 1.10)
Yes (n=7160)	2274 (31.8)	1.04 (0.97 to 1.12)	1.13 (1.04 to 1.22)**

***p<0.001; **p≤0.01.

IMD, index of multiple deprivation; 5, most deprived, 1, least deprived model 1 is a simple binary logistic regression with having received physical activity (PA) advice (Y/N) as the dependent factor. Model 2 is a multivariable binary logistic regression model adjusting for all sociodemographic and treatment factors. ‘All other’ treatment factors (been treated but still present/has not been treated/has come back/not certain).

**Figure 1:**
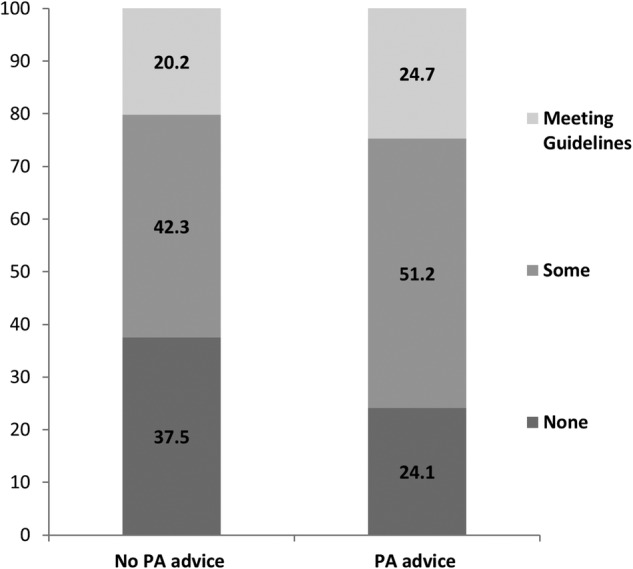
Proportions of patients doing at least some activity, or meeting the guidelines by advice. Values are from n=15 254 colorectal cancer patients who reported the number of days brisk physical activity (PA) was carried out in the past week as none, some (1–4 days) or meeting guidelines (5–7 days). χ^2^ value for difference s262.822; p<0.001.

Consistent with our hypothesis, recalling being given PA advice was associated with higher levels of current activity. Fifty-one per cent of patients who had been given advice were doing at least some activity, with 25% meeting the guidelines, compared with 42% and 20%, respectively, in the ‘no advice’ group (see table 3 and figure 1). Compared with the ‘no advice’ group, the odds of doing some activity were 1.88 (CI 1.74 to 2.05; p<0.001) and the odds of meeting guidelines were 1.90, (CI 1.75 to 2.09; p<0.001) among those who were given advice.

**Table 3:** Associations between recall of being given physical activity advice, demographic factors and activity levels in 15 254 patients with colorectal cancer

PA level (reference ‘None’)	Model 1 (unadjusted) OR (95% CI)	Model 1 (unadjusted) OR (95% CI)	Model 2 (adjusted) OR (95% CI)	Model 2 (adjusted) OR (95% CI)
	Some PA	Meeting guidelines	Some PA	Meeting guidelines
PA advice given				
No (n=10 520)	1	1	1	1
Yes (n=4734)	1.88 (1.74 to 2.05)***	1.90 (1.75 to 2.09)***	1.74 (1.60 to 1.90)***	1.70 (1.54 to 1.88)***
Sex				
Female (n=6091)	1	1	1	1
Male (n=9163)	1.11 (1.03 to 1.20)***	1.75 (1.60 to 1.92)***	1.04 (0.96 to 1.12)	1.63 (1.49 to 1.80)***
Age				
>85 (n=777)	1	1	1	1
75–84 (n=3797)	2.21 (1.85 to 2.66)***	2.20 (1.76 to 2.76)***	2.21 (1.84 to 2.65)***	2.15 (1.71 to 2.70)***
65–74 (n=5680)	4.11 (3.44 to 4.91)***	3.62 (2.90 to 4.52)***	4.05 (3.38 to 4.85)***	3.43 (2.74 to 4.29)***
55–64 (n=3677)	4.64 (3.86 to 5.57)***	3.64 (2.89 to 4.78)***	4.48 (3.72 to 5.40)***	3.42 (2.74 to 4.29)***
<55 (n=1323)	4.30 (3.49 to 5.30)***	3.06 (2.34 to 3.98)***	4.22 (3.41 to 5.21)***	3.00 (2.31 to 3.92)***
IMD				
5 (n=1606)	1	1	1	1
4 (n=2441)	1.17 (1.01 to 1.34)	1.17 (0.99 to 1.39)	1.22 (1.06 to 1.41)**	1.23 (1.04 to 1.47)
3 (n=3335)	1.35 (1.18 to 1.54)***	1.24 (1.05 to 1.45)	1.41 (1.23 to 1.61)***	1.30 (1.10 to 1.53)**
2 (n=3844)	1.68 (1.47 to 1.92)***	1.46 (1.25 to 1.72)***	1.75 (1.53 to 2.00)***	1.53 (1.30 to 1.79)***
1 (n=4028)	1.85 (1.62 to 2.11)***	1.52 (1.30 to 1.79)***	1.95 (1.71 to 2.24)***	1.61 (1.37 to 1.89)***
Ethnicity				
White (n=14 712)	1	1	1	1
All other (n=542)	1.01 (0.83 to 1.23)	1.04 (0.82 to 1.32)	1.00 (0.82 to 1.23)	1.02 (0.81 to 1.30)

***p<0.001; **p≤0.01.

IMD, index of multiple deprivation, 1, least deprived, 5, most deprived. Model 1 simple multinomial logistic regression with PA none, some (>30 min brisk activity on 1–4 days) and meeting guidelines (5–7 days) as the dependent variable. Model 2 adjusts for sex, age, IMD and ethnicity. ‘All other’ treatment factors (been treated but still present/has not been treated/has come back/not certain).

There were associations between demographic variables and activity levels (men, younger patients and those from higher SES backgrounds were more active; table 2), but associations between PA advice and activity levels remained significant after controlling for these and the adjusted odds were similar to the unadjusted values (OR 1.74, CI 1.60 to 1.90, p<0.001 for doing some activity; OR 1.70, CI 1.54 to 1.88, p<0.001 for meeting guidelines).

Similarly, although some health and treatment factors were related to activity levels (table 4), the association between PA advice and current activity levels remained after adjustment for treatment and presence of any LTC.

**Table 4:** Associations between being given physical activity (PA) advice, treatment and activity levels 2–3 years later in 15 254 patients with colorectal cancer

PA level (reference ‘none’)	Model 1 (unadjusted) OR (95% CI)	Model 1 (unadjusted) OR (95% CI)	Model 2 (adjusted) OR (95% CI)	Model 2 (adjusted) OR (95% CI)
	Some PA	Meeting guidelines	Some PA	Meeting guidelines
PA advice given				
No (n=10 520)	1	1	1	1
Yes (n=4734)	1.88 (1.74 to 2.05)***	1.90 (1.75 to 2.09)***	1.76 (1.61 to 1.91)***	1.79 (1.56 to 1.92)***
Time since treatment				
Still having (n=289)	1	1	1	1
<1 year (n=1938)	1.34 (1.02 to 1.76)	1.26 (0.89 to 1.77)	1.06 (0.79 to 1.41)	0.96 (0.67 to 1.37)
≥1 year (n=12 715)	1.59 (1.22 to 2.05)***	1.57 (1.14 to 2.17)***	1.22 (0.93 to 1.60)	1.14 (0.82 to 1.60)
Unknown (n=312)	0.78 (0.50 to 1.22)	0.78 (0.49 to 1.22)	1.07 (0.73 to 1.54)	1.03 (0.64 to 1.63)
Treatment response				
All other (n=3228)	1	1	1	1
In remission (n=12 026)	1.89 (1.73 to 2.06)***	2.15 (1.92 to 2.40)***	1.65 (1.50 to 1.81)***	1.90 (1.69 to 2.13)***
Long-term condition				
Yes (n=7160)	1	1	1	1
Unknown (n=2099)	1.22 (1.10 to 1.37)***	1.42 (1.24 to 1.62)***	1.28 (13.14 to 1.43)***	1.49 (1.30 to 1.71)**
No (n=5995)	1.88 (1.74 to 2.04)***	2.24 (2.03 to 2.47)***	1.73 (1.59 to 1389)***	2.17 (1.97 to 2.40)***

***p<0.001.

Model 1 simple multinomial logistic regression with PA none, some (>30 min brisk activity on 1–4 days) and meeting guidelines (>30 min brisk activity on 1–4 days) as the dependent variable. Model 2 adjusts for treatment, long-term condition, sex, age and IMD. All other treatment factors (been treated but still present/has not been treated/has come back/not certain).

## Discussion

To the best of our knowledge, this was the largest population-based study to explore the extent to which patients with cancer are given PA advice, and the only one to examine associations between recall of PA advice and activity levels in patients with CRC. Consistent with findings from other studies,^8–11^ a relatively small proportion recalled being given PA advice. In support of our hypothesis, recall of advice was associated with higher activity levels, even after adjustment for sociodemographics and treatment factors.

The finding that being given advice related to later activity levels in patients with CRC is supported by evidence from a small RCT in breast cancer survivors, which found a clear benefit from clinician advice over usual care in the short term.^16^ Our results also concur with an earlier survey which found that only 28% of cancer survivors (predominantly breast and prostate) reported that the oncologist had initiated any discussion around PA, but that this discussion was associated with higher activity levels during treatment.^7^ Our results strengthen the case for clinicians to recommend PA to their patients with cancer. In the ‘advice’ group, the proportion of participants doing at least some activity was 10% higher than in the ‘no advice’ group, and those meeting the PA guidelines 5% higher. This difference is potentially of real practical significance.^14^

Less than a third (31%) of our large sample of CRC survivors recalled having received PA advice. Women, older patients and those from lower SES backgrounds were less likely to recall having been given advice. Older patients were also less likely to have received advice in a Canadian survey, which the authors speculated could be due to them being less likely to initiate discussions about PA,^7^ although nearly all patients expressed a wish for their oncologist to initiate the discussion.^7^ Clinicians may want to consider whether these populations need more targeted advice to help make access to important lifestyle advice more equitable.

Giving PA advice may not always be easy for healthcare professionals. A recent qualitative study with cancer specialists identified lack of appropriate support as a barrier to discussing lifestyle with patients with cancer.^23^ This may be particularly applicable when discussing PA with vulnerable groups, such as older patients and those from lower SES groups. The same study also found that some specialists did not believe that lifestyle change would influence cancer risk and cited this as a reason for not discussing it. In a recent survey completed by 323 CRC clinicians in Scotland, only half (52%) reported being familiar with the lifestyle advice for patients with CRC and many felt they lacked the skills, confidence and knowledge to discuss it.^24^ In line with this, a survey of 274 oncology nurses found being ‘unsure what to recommend’ was strongly related to whether they gave advice or not.^25^ Insufficient time during the consultation was identified as a barrier in another study, along with unclear recommendations.^8^ These observations indicate a need to address the gaps in knowledge and skills while keeping in mind the time constraints in consultations. Finally, the evidence that PA reduces risk of recurrence and cancer-specific mortality in CRC currently comes from observational cohorts;^1^ results of an RCT examining PA effects on survival of CRC (CHALLENGE)^26^ are not yet available, so clinicians may not yet be convinced to recommend PA for survival outcomes. However, in light of strong evidence for a number of other important outcomes, such as reductions in cancer-related fatigue and improved quality of life,^3^ it is important for clinicians to be advising their patients with CRC to be physically active.

This study had limitations. Data were self-reported using single item measures, so PA may have been under-estimated or over-estimated. Additionally, no information was gathered on who gave the advice and when in the care pathway. This study was based on secondary analyses of existing large-scale PROMS data and provides an important ‘first step’. Future large-scale surveys in cancer survivors should use validated measures of PA and collect detailed information on the context of the delivery of the PA advice. Information on receiving PA advice depended on patient recall and therefore, could also be under-estimated or over-estimated. Consistency with findings from other studies, both from the patient and the health professional perspective, is reassuring.^7^ ^9–11^ It is also important to note that recall of advice is an important outcome in itself in this context.^16^

The possibility that those recalling PA advice were already more aware of the importance of PA or had higher pretreatment levels cannot be ruled out, and longitudinal studies are warranted. However, this could also suggest the need for research on how to make important messages about PA more salient to those who are less active and less engaged. One RCT in the field suggested that clinician's brief exercise advice increased subsequent activity levels of breast cancer survivors^16^ and though this is very promising, trials in other cancers and with long-term follow-ups are required. The use of patient-reported disease and treatment variables should also be viewed with caution. Additionally, the sample were predominantly white, older and in remission, which means that the findings cannot be generalised to other populations. A further limitation is the lack of data on cancer staging, because patients with more advanced disease may be less likely to have been given advice or to be doing any PA. In clinician surveys, disease stage has not been raised as a major concern; rather the lack of knowledge and time are cited as key barriers.^25^

## Conclusion

Recalling being given PA advice after a diagnosis of CRC was associated with higher levels of PA. However, less than a third of patients recalled receiving advice. Future research should examine the context in which advice is given. However, encouraging clinicians working with CRC patients to give brief PA advice is warranted and may help improve outcomes for CRC survivors.
